# Probing the Effect of Bulky Lesion-Induced Replication Fork Conformational Heterogeneity Using 4-Aminobiphenyl-Modified DNA

**DOI:** 10.3390/molecules24081566

**Published:** 2019-04-20

**Authors:** Ang Cai, Ke Bian, Fangyi Chen, Qi Tang, Rachel Carley, Deyu Li, Bongsup P. Cho

**Affiliations:** Department of Biomedical and Pharmaceutical Sciences, College of Pharmacy, University of Rhode Island, 7 Greenhouse Road, Kingston, RI 02881, USA; angcai@uri.edu (A.C.); kebian@uri.edu (K.B.); fangyi_chen@uri.edu (F.C.); qitang@my.uri.edu (Q.T.); rachel_carley@my.uri.edu (R.C.)

**Keywords:** 4-aminobiphenyl, bulky DNA lesion, conformational heterogeneity, surface plasmon resonance (SPR) binding kinetics, steady state enzyme kinetics, Klenow fragment

## Abstract

Bulky organic carcinogens are activated in vivo and subsequently react with nucleobases of cellular DNA to produce adducts. Some of these DNA adducts exist in multiple conformations that are slowly interconverted to one another. Different conformations have been implicated in different mutagenic and repair outcomes. However, studies on the conformation-specific inhibition of replication, which is more relevant to cell survival, are scarce, presumably due to the structural dynamics of DNA lesions at the replication fork. It is difficult to capture the exact nature of replication inhibition by existing end-point assays, which usually detect either the ensemble of consequences of all the conformers or the culmination of all cellular behaviors, such as mutagenicity or survival rate. We previously reported very unusual sequence-dependent conformational heterogeneities involving FABP-modified DNA under different sequence contexts (TG_1_*G_2_T [67%B:33%S] and TG_1_G_2_*T [100%B], G*, *N*-(2′-deoxyguanosin-8-yl)-4′-fluoro-4-aminobiphenyl) (Cai et al. *Nucleic Acids Research*, 46, 6356–6370 (2018)). In the present study, we attempted to correlate the in vitro inhibition of polymerase activity to different conformations from a single FABP-modified DNA lesion. We utilized a combination of surface plasmon resonance (SPR) and HPLC-based steady-state kinetics to reveal the differences in terms of binding affinity and inhibition with polymerase between these two conformers (67%B:33%S and 100%B).

## 1. Introduction

**Figure 1:**
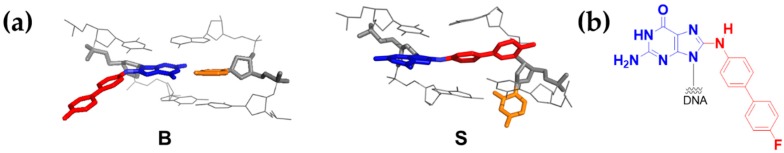
(**a**) Two major conformational views of dG-C8-FABP [*N*-(2′-deoxyguanosin-8-yl)-4′-fluoro-4-aminobiphenyl]: B-, and S-conformers. Fluorine-labeled ABP (FABP)-red, modified dG-blue, complementary C-orange. (**b**) The chemical structure of dG-C8-FABP.

The human genome is under constant assault from the environment. DNA-damage-induced mutations are known to trigger chemical carcinogenesis [1,2,3,4,5]. Therefore, understanding the biological responses of cells to DNA mutations is important. Arylamines belong to a notorious group of environmental mutagens/carcinogens that produce bulky DNA adducts in vivo [6,7,8,9,10]. They are known to adopt different unique conformational motifs: the major groove B-type (B), stacked (S) (Figure 1a) [11], or the minor groove wedge (W) [12]. Consequently, these bulky-lesion induced conformational heterogeneities complicate mutational and repair outcomes [9,10,11,13,14,15]. 

4-Aminobiphenyl (ABP) is a major etiological agent of human bladder carcinoma and a potent urinary-bladder carcinogen in experimental animals. As such, commercial production of ABP is banned, however, exposure to ABP can still take place from cigarette smoke. ABP is activated by cellular *N*-acetyltransferase to produce a dG-C8-substituted adduct as a major DNA lesion (dG-C8-ABP, Figure 1b) [12,16,17,18,19,20]. The majority of human bladder cancer has a mutation in the p53 gene. Compared with other cancers, the ABP-induced mutations are more evenly distributed along the p53 gene and the mutation hotspots occur at both CpG, such as codons 175, 248, and 273, and non-CpG sites, such as codons 280 and 285, the latter two being unique mutational hotspots for bladder and other urinary tract cancers [21]. The major induced mutation is a G to T transversion mutation. Translesion synthesis (TLS) over dG-C8-ABP in two different sequences (CCG*GAGGC and CCGGAG*GCC, G* = dG-C8-ABP), which represent the codon 248 and 249 sequences of the human p53 tumor suppressor gene, respectively, has confirmed that codon 248 is both a hot spot of ABP adduct formation and G to T mutation [22]. These results suggest that the efficiency of TLS over dG-C8-ABP is influenced by the surrounding DNA sequences. The structurally similar liver carcinogens, 2-aminofluorene (AF) and *N*-2-acetylaminofluorene (AAF), produce similar C8-dG adducts. AF could be error-free by correctly base pairing with an incoming dC, whereas AAF blocks the replication process and requires recruitment of lesion bypass polymerases for TLS. In *Escherichia coli*, the TLS of AF produces point and frameshift mutations, whereas bypass of the AAF lesion is frequently accompanied by a frameshift mutation. 

Our previous structural studies showed that fully paired duplexes containing the fluorine containing model dG-C8-FABP (fluorine-labeled ABP, FABP, *N*-(2′-deoxyguanosin-8-yl)-4′-fluoro-4-aminobiphenyl) adopted a 67%:33% mixture of the B- and S-conformers in the TG_1_*G_2_T (G* = FABP) sequence context at 25 °C [23]. Meanwhile, the same lesion in the TG_1_G_2_*T context exhibited exclusively the B-type conformation under identical experimental conditions. When a replication polymerase encounters a bulky DNA lesion, the polymerase is likely to stall, thus stopping DNA synthesis. The lesion also influences whether the DNA replication will be error-free or error-prone. The aforementioned striking sequence effect in buffer systems warrants systematic studies in the presence of a polymerase. Vaidyanathan et al. [24] probed the sequence effect of dG-C8-AF (AF, aminofluorene), a structural analog of ABP, on nucleotide insertion efficiencies catalyzed by the Klenow fragment (Kf-exo^−^) on TG*A and CG*A sequences [25,26]. They found that the S conformer of CG*A thermodynamically favors the insertion of mutagenic A over non-mutagenic C at the lesion site. Xu et al. reported that Kf-exo^−^ is a strong binder to template/primer junctions, but with minimal nucleotide selectivity against modified DNA [27]. The sequence-dependent conformational heterogeneity may play an important role in DNA replication and mutation. We hypothesize that different conformations lead to different extents of polymerase binding affinities, kinetic behaviors, and replication blocks, ultimately resulting in complex toxic and mutational outcomes. Here we conducted surface plasmon resonance (SPR) binding experiments and steady-state enzyme kinetics in vitro to probe the effect of ABP-induced conformational heterogeneity on DNA replication. 

## 2. Results

### 2.1. DNA Sequence Systems

**Figure 2:**
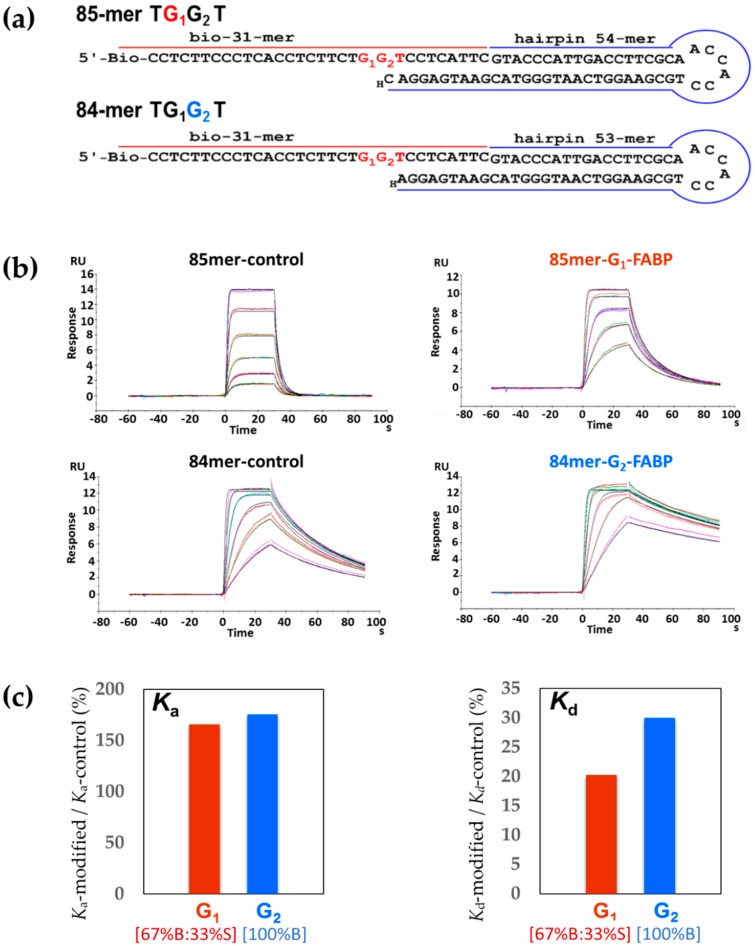
(**a**) Hairpin template−primer oligonucleotide constructs of 85-mer G_1_ and 84-mer G_2_ for Kf-exo^−^, H denotes 3′-dideoxy-nucleotide; (**b**) Sensorgrams of binary complexes of Kf-exo^−^ with 85-mer and 84-mer unmodified control, G_1_-FABP and G_2_-FABP modified sequences (1:1 binding fitted curves are overlaid as black lines); (**c**) *K*_a_ (*K*_a_-modified/*K*_a_-contol) and *K*_d_ (*K*_d_-modified/*K*_d_-contol) ratio, G_1_ and G_2_ represent the ratio of 85-mer FABP-G_1_/85-mer-control and 84-mer FABP-G_2_/84-mer-control, respectively.

For SPR binding experiments, we constructed two biotinylated hairpin-based template–primer strands (Figure 2a), 85-mer G_1_* adduct and 84-mer G_2_* adduct (G* = dG-C8-FABP). Specifically, two FABP-modified–biotin–31-mer DNA sequences (TG_1_*G_2_T and TG_1_G_2_*T) were purified by HPLC and characterized individually by enzyme digestion/MALDI-MS [27,28,29,30] (Figure S1). A 54-mer hairpin DNA was annealed and ligated to the biotin–31-mer TG_1_*G_2_T oligonucleotide, whereas a 53-mer hairpin DNA was ligated to the biotin–31-mer TG_1_G_2_*T oligonucleotide. The hairpin structures were used to improve the thermal stability of the oligonucleotide duplex during SPR experiments and the lesion G_1_* and G_2_* adduct were placed at the 21st and 22nd bases, respectively, to avoid a clash between the polymerase protein and the gold chip surface [27,31,32].

**Figure 3:**
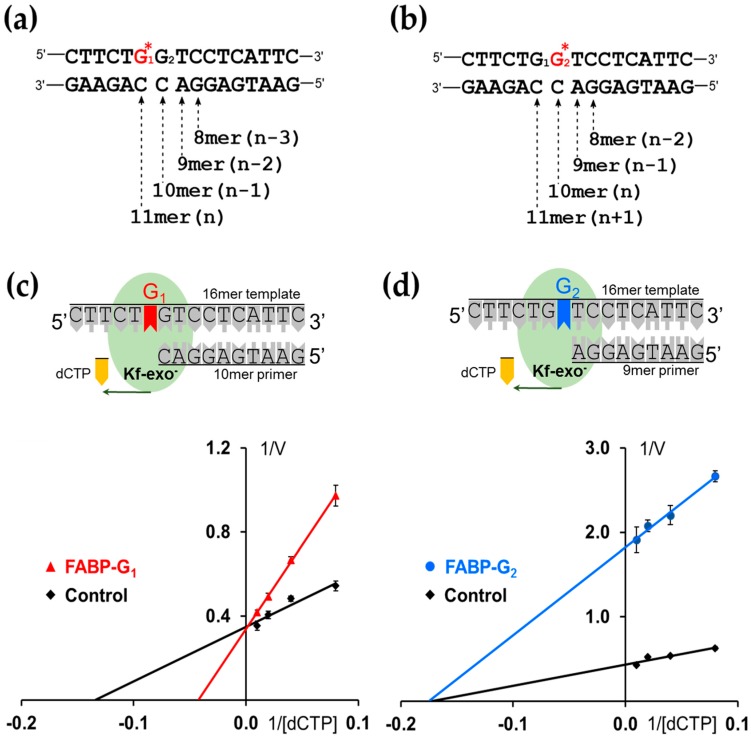
DNA replication models for (**a**) FABP-modified G_1_* (TG*GT) templates and (**b**) FABP-modified G_2_* (TGG*T) templates. n: lesion site. Lineweaver–Burk model of DNA synthesis catalyzed by Kf-exo^−^ at (**c**) 10-mer, G_1_-FABP and (**d**) 9-mer G_2_-FABP lesion sites with dCTP at steady-state. The G_1_-FABP against with Kf–exo^–^ adopts a competitive inhibitor model, whereas the behavior of TG_1_G_2_*T shows as non-competitive (see text).

For HPLC-based steady-state kinetics experiments, two 16-mer G_1_* or G_2_* template strands were each annealed to various lengths of complementary strands (8-mer to 11-mer) to create n − 3, n − 2, n − 1, and n for the G_1_* adduct and n − 2, n − 1, n, and n + 1 for G_2_* (n is the lesion site) (Figure 3a,b) [23,33,34,35].

### 2.2. Oligonucleotide Characterization by MALDI-TOF MS (Matrix-Assisted Laser Desorption/Ionization-Time of Flight Mass Spectrometry)

The FABP-modified biotin–31-mer oligonucleotides were characterized by exonuclease enzyme digestion followed by MALDI-TOF MS analysis in accordance with the published procedures [27,36]. In the present case, 5′-3′ exonuclease digestion on DNA was difficult to carry out due to the binding hindrance of the 5′-biotin motif to the enzyme. Figure S1b shows the MALDI-TOF MS spectra of the 3′-5′ snake venom phosphodiesterase (SVP) exonuclease digestions of the biotin–31-mer G_1_* adduct at different time points (0, 1, 4, 6, 7, 8, and 10 min). The *m/z* of 9804 (theoretical 9803) at 0 min represents the control mass-to-charge ratio. Within 6 min of 3′–5′ exonuclease digestion, the lower masses appeared corresponding to the 27-mer to 21-mer fragments. The *m/z* = 6788 (theoretical 6787) fragment, which persisted from 6 min to 10 min was assigned to the G_1_*-FABP-modified 21-mer. These results confirm the first eluting peak (peak 1) from the HPLC profile (Figure S1a) is biotin–31-mer TG_1_*G_2_T. Figure S1c presents the MALDI-TOF MS spectra of the peak 2 on HPLC with 3′–5′ exonuclease digestions. The digestions were fast in the first 4 min showing the *m/z* range from 9803 to 7116. However, the digestion stalled from 4–10 min at *m/z* 7116, which corresponds to the 22-mer fragment containing the FABP-modified guanine (theoretical *m/z* = 7116). These results confirm that peak 2 is G_2_*-FABP-modified biotin–31-mer. 

The 84- and 85-mer biotinylated oligonucleotides were purified and identified by 15% denaturing polyacrylamide gel (Bio-rad, Hercules, CA, USA) [27]. Figure S2 reveals the denaturing gel profiles of unmodified/modified 84- and 85-mer ligated oligonucleotides, biotin–31-mer, and 53-mer/54-mer non-ligated oligonucleotides. For the 85-mer control, all the biotin–31-mer and 54-mer hairpins were ligated. As for the 84-mer control, 85-mer G_1_*, and 84-mer G_2_*, excessive biotin–31-mer, 54-mer, and 53-mer were observed correspondingly. The ligated and purified 85-mer control/ G_1_* and 84-mer control/ G_2_* were used for SPR experiments. 

### 2.3. HPLC-Based Steady-State Kinetics

We conducted steady-state experiments to investigate the impact of conformational heterogeneity on nucleotide insertion kinetics [37]. The *E. coli* exonuclease-deficient Kf-exo^−^ was used for single-nucleotide incorporation. Although the modified base could pair with dCTP to complete the primer extension reaction induced by Kf-exo^−^, the reaction efficiency was much poorer than the regular DNA template. The change of efficiency is represented by the enzyme kinetic parameters, K_m_ and k_cat_, and the results are summarized in Table 1. The bulky C8 adduct on guanine does not directly block the Watson–Crick base pairing, but it could either physically interfere with the dNTP binding pocket in Kf-exo^−^ when the FABP-G holds an “S” conformation; or distort the Kf-exo^−^ structure in the ternary complexes and influence the geometry at the active site of forming phosphodiester bonds when FABP-G holds the “B” conformation (Figure 1a). In both scenarios, the bulky adduct acts as an inhibitor, but in two different ways (Figure 3). In order to apply the inhibition kinetic model, the whole primer extension assay was performed by maintaining the concentration of inhibitor (FABP-containing DNA duplex), and varying the concentration of substrate, dNTP (dCTP or dATP). dATP was added to 16/8-mer and 16/11-mer systems, whereas dCTP was added to the 16/9-mer and 16/10-mer sequences (Figure 3). 

### 2.4. dCTP Incorporation

Specifically, adding dCTP to the n − 1 (16/10-mer) G_1_* adduct elongated a single 11-mer (Figure 3a), whereas the n − 1 (16-/9-mer) G_2_* adduct produced 10- and 11-mer (Figure 3b). The formation of 10- and 11-mer mixture from the G_2_* adduct sequence was difficult to quantify; thus, the reduction of the starting material 9-mer was employed to determine the kinetic parameters for both G_1_* and G_2_* reactions.

The n − 1 (16/9-mer) G_2_* adduct showed a *K*_m_ value (5.7 µM) similar to that of the control (5.8 µM); such similarity indicates an almost equal affinity (Table 1). However, the insertion efficiency *f*_ins_ of dCTP opposite −G_2_* was 4-fold lower than that of the unmodified control (0.24:1) (Table 1). By contrast, the *K*_m_ of the G_1_* adduct showed a lower affinity (23.4 µM) than that of the control (7.4 µM). The insertion efficiency *f*_ins_ in G_1_* was three times lower than the control (0.33 to 1). These two forms of enzyme inhibition can be clearly shown by the Lineweaver–Burk plot. As shown by the Lineweaver–Burke enzyme inhibition model (Figure 3c), the (16/10-mer) G_1_* adduct and its control intersect on the Y-axis and a competitive inhibition behavior is suggested. By contrast, the n − 1 16/9-mer G_2_* adduct and its control merge at the x-axis. This behavior can be explained as non-competitive inhibition (Figure 3d). When the n − 1 (16/9-mer) G_2_* adduct elongated to 10-mer (n) and 11-mer (n + 1), few 10-mer primers, but most of the 11-mer primers, were observed from the HPLC profile. This result was achieved because the bulky FABP on the G_2_ at the lesion site (n) does not block the replication from n − 1 to n + 1.

### 2.5. dATP Incorporation

The long-range lesion effect was examined by initiating the primer elongation with a 16/8-mer template/primer that produces n − 2 for G_2_* (Figure 3b) and n − 3 for G_1_* (Figure 3a). Figure S3a presents the results at 10 min. In the control, ~70% of the 8-mer was extended to 9-mer. With the adduct on G_1_, ~40% of the 8-mer was converted to 9-mer, indicating a moderate pre-lesion effect at G_1_*. However, G_2_* blocked ~80% of the 8-mer converting to 9-mer, which indicates a much stronger pre-lesion effect than that of G_1_*. These results are not surprising because the 8-mer primer is closer to the G_2_* adduct than the G_1_* adduct. In the 11-mer (Figure S3b), the unmodified control exhibited 90% of dATP insertion. However, only ~20% of the 11-mer was elongated to 12-mer opposite the G_1_* lesion. This finding indicates a strong post-lesion effect. Moreover, only ~55% of the 11-mer was converted to 12-mer in the n + 1 for G_2_*. These results suggest significant retardation of insertion close to the lesion site. 

### 2.6. Kf-exo^−^ SPR Binding Kinetics

Binary System—Figure 2b shows the sensorgrams for the binary binding between Kf-exo^−^ and unmodified controls (85-mer/84-mer TGGT) or modified sequences (85-mer TG_1_*G_2_T/84-mer TG_1_G_2_*T). These sequences represent replication forks for the G_1_* and G_2_*, respectively. Kf-exo^−^ showed a much stronger binding affinity (*K*_D_) to the modified 85-mer TG_1_*G_2_T (8.3-fold) and 84-mer (5.6-fold) TG_1_G_2_*T sequences relative to their unmodified controls, 85-mer and 84-mer TGGT, respectively (Table 2). The differences were more striking in the dissociation rates (85-mer TGGT *k*_d_: 0.84 s^−1^ vs. 85-mer TG_1_*G_2_T *k*_d_: 0.17 s^−1^; 84-mer TGGT *k*_d_: 0.03 s^−1^ vs. 84-mer TG_1_G_2_*T *k*_d_: 0.009 s^−1^). There was a substantial difference in the *K*_d_ ratio (*K*_d_-modified/*K*_d_-control) between G_1_ and G_2_ (Figure 2c). By contrast, the *K*_a_ ratios of G_1_ and G_2_ were close to each other, indicating similar association rates. The stabilization energies here specifically measure the energies of forming hydrogen bonds between Kf-exo^−^ and DNA sequences [38]. For the binary system the net stabilization energies were positive (Table S1), which indicates the hydrogen bonds that form between Kf-exo^−^ and modified sequences (85-mer TG_1_*G_2_T/84-mer TG_1_G_2_*T) are stronger than their unmodified controls (85-mer/84-mer TGGT). It is interesting to note that although only one base length (dC) differed between the 84-mer and 85-mer, the binding affinity of the unmodified 84-mer was 18.2-fold tighter than that of the 85-mer. Similarly, the *K*_D_ of the 84-mer TG_1_G_2_*T was 12.2-fold greater than that of the 85-mer TG_1_*G_2_T. 

**Figure 4:**
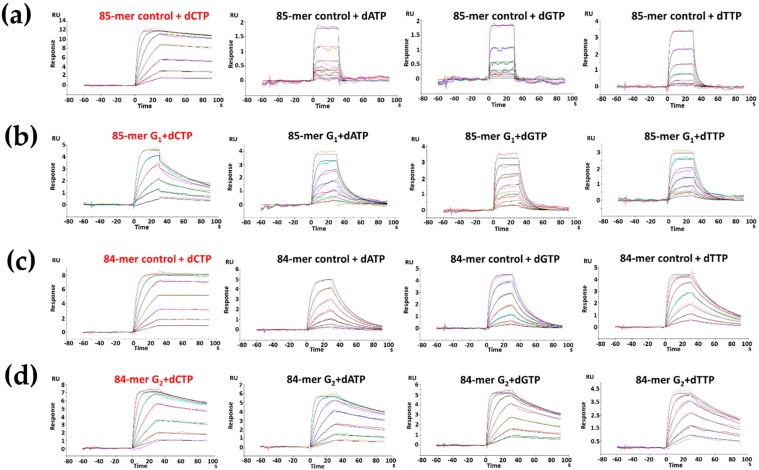
Sensorgrams of the ternary Kf-exo^−^ complexed with (**a**) 85-mer control, (**b**) 85-mer TG_1_[FABP]G_2_T, (**c**) 84-mer control and (**d**) 84-mer TG_1_G_2_[FABP]T sequences in the presence of dNTPs (1:1 binding fitted curves are overlaid as black lines).

Ternary System— dNTP was added to form Klenow ternary complexes. Kf-exo^−^ bound tightly to the unmodified controls when the correct dCTP was introduced in the ternary system (Figure 4). In the unmodified 85-mer, the binding affinities of dATP, dGTP, and dTTP to the 84-mer controls were reduced by 315-, 284-, and 89-fold, respectively, relative to that of dCTP (Table 2). However, nucleotide selectivity was significantly decreased for the ternary complexes with FABP-dG adduct. The *K*_D_ value for the 85-mer TG_1_*G_2_T was only reduced by ~5-fold in dATP, dGTP, and dTTP compared to that of dCTP. Similar results were obtained for the 84-mer G_2_* adduct, where binding tightness was reduced by ~2-fold in dATP, dGTP, and dTTP compared with that of dCTP. Moreover, as in the binary system, slower dissociation rates were observed for the unmodified and G_2_* modified 84-mers. The *K*_D_ values of dCTP, dATP, dGTP, and dTTP in the unmodified and G_2_* modified 84-mers were smaller than those of the unmodified 85-mer (5- and 13-fold smaller in dCTP and dTTP) and G_1_* modified 85-mer TG_1_*G_2_T (4-, 13-, 10-, and 12-fold smaller in dCTP, dATP, dGTP, and dTTP, respectively). 

## 3. Discussion

We previously conducted [23] systematic spectroscopic, thermodynamic, and chip binding (^19^F-NMR, CD, DSC, and SPR) studies for the extension of 16-mer TG_1_*G_2_T and TG_1_G_2_*T (G* = FABP) sequences. These protein free model systems mimic a translesion synthesis of the bulky FABP lesion in two very distinctive sequence-dependent conformational heterogeneities at G_1_ (67%B:33%S) and G_2_ (100%B). The results indicate that the sequence-dependent conformational complexities appear to persist at various elongation positions, including the ss/ds junction. The B-conformer is a major thermodynamic stabilizer in duplex settings, whereas the S-conformer is a destabilizer. However, the opposite is the case for the adduct at the ss/ds junction. In particular, the S-conformation promotes lesion stacking with nascent base pairs at the replication fork. SPR results reveal that the S-conformation increases the binding affinity with the complementary strands in the order of G_1_* > G_2_*. In the present work, we examined the effects of these unusual sequence effects on Klenow polymerase binding (binary and ternary) and nucleotide insertion kinetics.

### 3.1. Improvement on Model Hairpin Oligonucleotide Construction

We previously reported a construction of a biotinylated hairpin-based template–primer strand for DNA–polymerase SPR binding studies [27,39,40]. The general strategy was to ligate a biotinylated arylamine-modified 31-mer sequence with 52-mer hairpin DNA to form an 83-mer hairpin. This was then followed by addition of a ddNTP to the 3′-end to prevent potential primer elongation. However, the ddNTP addition step was low yielding and the purification of ligated products was difficult. In the present study, we succeeded in direct ligation of a biotinylated modified 31-mer with ddNTP-containing 53- and 54-mer hairpin DNA. This process improved the yield significantly (from ~10% to ~ 30%), and the products were readily separated by HPLC. Also, previously, individual dNTPs (100 μM) were mixed with varying concentrations of Kf-exo^–^ in sample buffers and injected over the surface without any dNTP in the running buffer. This would create a complication resulting in two variables, concentrations of dNTPs and Kf–exo^–^ [39]. In the present work, we circumvented the complication by adding individual dNTP in running buffer and introducing Kf–exo^–^ directly onto the chip surface. This system has only one variable, thus increasing the system stability and accuracy for the translesion synthesis. 

### 3.2. Lesion and Sequence Effects on SPR Binding Affinities and Kinetics

Tight binding of Kf–exo^–^ with the unmodified dG control was observed when the correct nucleotide dCTP was presented in both the 85-mer and 84-mer. This result is in a good agreement with expectation and shows high nucleotide selectivity (*K*_D dCTP_ ≪ *K*_D dTTP_ < *K*_D dATP_ ~ *K*_D dGTP_). Meanwhile, a remarkably tighter binding of Kf–exo^–^ was found for the FABP-modified sample relative to the control: i.e., the *K*_D_ of the 85-mer G_1_* and 84-mer G_2_* interactions were 8.3 and 5.6 fold higher than the corresponding unmodified controls, respectively. Adduct-induced tighter binding affinity with Kf–exo^–^ has been reported [27,41] and may be due to the interactions between the bulky FABP and the nearby hydrophobic amino-acid residues in the active site of the Kf-exo^–^. In the ternary system, nucleotide selectivity is low. In particular, the *K*_D_ of the dCTP at the opposite G_1_* and G_2_* are only ~5- and ~2-fold tighter than the incorrect dATP, dGTP, and dTTP, respectively. This is a strong lesion effect. We observed that the usual 1:1 model SPR simulation did not provide a clean fit of dCTP for G_1_* in the ternary system. It is possible that the present DNA adduct-Kf complex exists in multiple stages due to the FABP-induced S/B conformational heterogeneity at the G_1_* replication fork. However, in most cases, conformational changes in proteins are much faster than the SPR time scale and thus additional studies would be necessary to confirm these possibilities.

Our HPLC-based steady-state kinetics data indicate competitive inhibition for the S/B-conformer replication fork at G1*. In other words, the S/B conformational heterogeneity at G_1_* is inhibitory to replication probably due to some unfavorable clash of the bulky FABP lesion with the incoming dCTP in a competitive manner. The unfavorable interactions may be caused by the competition of the S-conformer with dCTP opposite of dG. However, the exclusively B-conformeric G_2_* accommodates well for the incoming dCTP, resulting in non-competitive inhibition. The equal affinity (*K*_m_) between G_2_* and its unmodified dG reveals that the B-conformation in the major grove may not interfere with the insertion of the correct dCTP. This finding might also explain the greater *K*_D_ of dCTP at G_1_* over G_2_* of the SPR results and indicates a weaker binding affinity for G_1_* over G_2_*. Alternatively, the B-conformer may not interfere with the Watson–Crick base pair, but it may alter the native conformations or hinder the formation of a phosphodiester bond. The S-conformer portion of G_1_* cannot provide a proper Watson–Crick base pair for replication and it may need to convert back to the B-conformation to be replicated properly. Thus, the S-conformer may function as a competitive inhibitor for replication. We observed a competitive-inhibition model for G_1_* but a non-competitive-inhibition model for G_2_*. These types of intermediate interactions may be a necessary step in the DNA polymerase proofreading process as well. 

## 4. Materials and Methods

### 4.1. Model Oligonucleotide DNA Sequences

FABP-modified 16-mer templates were prepared in accordance with the published procedures [42]: Two mono-adducts (d(5′-CTTCTG_1_*G_2_TCCTCATTC-3′)) (G* = FABP) (G_1_* adduct) and (d(5′-CTTCTG_1_G_2_*TCCTCATTC-3′)) (G_2_* adduct). 5′-Biotin-labeled 31-mer oligonucleotides, 5′-phosphorylated and 3′-dideoxy-A 53-mer, 5′-phosphorylated and 3′-dideoxy-C 54-mer were purchased from IDT (Integrated DNA Technologies Inc., Coralville, IA, USA) in desalted form and purified by reverse-phase high-performance liquid chromatography (RP-HPLC). Kf-exo^−^ (D355A, E357A) was purchased from NEB Inc. (Ipswich, MA, USA). All HPLC solvents were purchased from Fisher Inc. (Pittsburgh, PA, USA). The modified oligonucleotides were purified by HPLC (Thermo Scientific, Madison, WI, USA) on a Phenomenex Luna C18 analytical column (100 × 46 mm, 3.0 μm) (Phenomenex, Torrance, CA, USA). For the FABP-modified biotinylated 31-mer sequence, a 0.2 mL/min flow rate was used in a 48 min linear gradient profile (2% to 26%, *v/v*) acetonitrile with 100 mM TEAA buffer (pH 7.0) in mobile phase (Figure S1a). 

### 4.2. HPLC-based Steady-State Kinetic Analysis

The extension efficiency of Kf-exo^−^ polymerase opposite G_1_* and G_2_* adducts was determined by steady-state kinetic experiments as described by published procedures [27,43,44,45]. All reactions were performed using Kf-exo^−^ (100 nM) and primers (5 µM) in NEB buffer 2 (New England Biolabs, Ipswich, MA, USA). at 22 °C. The primers (8-mer to 12-mer) were pre-annealed with 6.75 µM FABP-modified or unmodified 16-mer templates by heating up to 70 °C and slowly cooling down to room temperature. Five different concentrations of dCTP or dATP (0, 12.5, 25, 50, and 100 µM) were used to initiate nucleotide insertion. All reactions were quenched by adding 10mM EDTA followed by immediately denaturing at 80 °C. The initial velocity of each reaction within the steady-state range was then obtained by performing every reaction within a short time period from 5 s to 30 s. The extended and unextended primers were separated by anion-exchange HPLC with a 1.5 M ammonium acetate gradient of 40–60% in water for 6.5 min and quantified by the absorbance of UV at 260 nm. All reactions were performed at 22 °C in triplicate, and the results were analyzed with a GraphPad Prism 5 software by using the Lineweaver–Burk model. The inhibition curves were fitted to the equation 1/*V* = 1/*V*_max_ + (*K*_M_/*V*_max_)/[S_dCTP_]. The relative insertion efficiency *f*_ins_ was obtained as (*k*_cat_/*K*_M_)_modified_/(*k*_cat_/*K*_M_)_unmodified_.

### 4.3. SPR Measurements

FABP-modified Hairpin Template/Primer Constructs. 5′-Biotinylated 31-mer containing dG-C8-FABP in the “-TG_1_G_2_T-” sequence context was used in SPR analysis following the reported procedures [27,39,40]. The two FABP-modified biotinylated 31-mer G_1_* and G_2_* adducts were separated by RP-HPLC and characterized by MALDI-TOF MS [36]. The 84-mer and 85-mer hairpin–template–primer were prepared by following the reported protocols [27,39]. Briefly, two different lengths of hairpin DNA sequences (53- and 54-mer) were phosphorylated at their 5′-ends, but their 3′-ends were modified with ddA and ddC, respectively, to prevent further primer elongation (Figure 2a). The biotinylated 31-mer modified G_1_* adduct and 54-mer hairpin were desalted by G-25 spin columns and annealed together by heating to 95 °C for 5 min and cooling to room temperature. The mixture solution was ligated in a buffer containing 4,000 units of T4 ligase enzyme for 16 h at room temperature. The resulting 85-mer oligonucleotide was purified in 15% denaturing polyacrylamide gel and extracted using the electroelution method followed by desalting by again using G-25 spin columns. The corresponding biotinylated 85-mer was finally purified by RP-HPLC. G_2_* adduct biotinylated 84-mer and unmodified biotinylated 84- and 85-mers were also prepared similarly. All the 84- and 85-mer oligonucleotides were identified by the 15% denaturing polyacrylamide gel (Figure S2).

Immobilization of Streptavidin (SA) on CM5 S Chip and DNA Coating. SPR experiments were carried out using Biacore T200 (GE Healthcare, Piscataway, NJ, USA). The SA via the amine coupling kit was immobilized on flow cells on the carboxymethylated dextran-coated CM5 S chip by following the reported procedures [27,39]. After SA immobilization at around 2000 RU on the flow cells, the chip surface was washed with 50 mM NaOH for 60 s five times to reach below 20 RU. Then, the running buffer was injected three times, and the system was equilibrated with running buffer for 2 h. The 84- and 85-mer unmodified and modified G_1_* and G_2_* biotinylated DNA sequences (2 nM) were injected over the flow cells (2 to 4) for 90–120 s to achieve 5-6 RU. The surface was stabilized with running buffer for 3 h before conducting SPR binding affinity experiments. 

### 4.4. Real-Time Kinetic Analysis by SPR

The SPR system was first primed at least three times with running buffer and zero-concentration injections to condition the plasmon surface. The DNA was coated on the SA surface of flow cell 2 to 4 (cell 1 as blank reference). Surface testing, regeneration buffer scouting, and the mass transport limitation test were conducted prior to kinetic experiments following previous reports [27,39]. DNA coating around 4–5 RU did not show any impact of mass transport. The steady-state affinity analysis of Kf-exo^−^ binding to unmodified and modified DNA was analyzed in the absence (binary) and presence of dNTPs (ternary) by varying Kf-exo^−^ concentrations.

For the binary system, Kf-exo^−^ was injected without dNTPs over the DNA surface in varying concentrations (0–25 nM) and repeated twice as described previously [27]. Briefly, the 1× HBS-P running buffer (containing 100 μg/mL bovine serum albumin [BSA] and 5 mM MgCl_2_) with six different concentrations Kf-exo^−^ (0–25 nM) was injected for 30 s at a flow rate of 100 μL/min over the cells. Afterward, 0.05% SDS was applied as regeneration solution at a flow rate of 100 μL/min over the surface for 30 s. The surface was stabilized with running buffer for 15 min after a regeneration step between different concentration circles. 

For the ternary system, dNTPs were injected with the HBS-P running buffer for comparison. 1× HBS-P running buffer (100 μg/mL BSA, 5 mM MgCl_2_, and 100 μM individual dNTPs) with varying concentrations of Kf-exo^−^ was injected over the surface. For both binary and ternary systems, sensorgrams were fitted by using a 1:1 Langmuir model, and the binding affinity constants (*K*_D_) were determined using steady-state affinity analysis in the BIA evaluation software v 1.0 provided by the manufacturer (GE Healthcare, Marlborough, MA, USA). 

## 5. Conclusions

In this paper, we conducted SPR and HPLC-based steady-state kinetics studies to probe adduct-induced conformation-dependent replication block during translesion synthesis (TLS). The FABP-modified DNA adduct adopts a mixture of B and S conformations in the TG_1_*G_2_T (67%B:33%S), but shows exclusively B conformation in the TG_1_G_2_*T sequence context [23]. According to the present Lineweaver–Burk enzyme inhibition model, the S/B-conformeric mix G_1_* adduct exhibits a competitive-inhibition, whereas the B-conformeric G_2_* adduct behaves as a non-competitive inhibitor in the nucleotide insertion step. These results indicate that the S-conformer may not be able to accommodate the incoming dCTP and exhibits a competitive behavior with incoming dNTPs, thus blocking replication. By contrast, the exclusive B-conformer G_2_* does not interfere with the Watson–Crick base pairing, resulting in a proper dCTP insertion. As such, the B-conformer shows a non-competitive-inhibition behavior. The SPR binding results implicate an adduct-induced tight binding with Kf-exo^−^ in a binary system. In the ternary system, nucleotide selectivity decreases when G_1_ and G_2_ are modified by FABP. From these experiments, we observed the effect of conformational heterogeneity induced by the bulky lesion on replication block. 

It has been reported that compared with other cancers, the ABP-induced mutations are more evenly distributed along the p53 gene and the mutation hotspots occur through the genome with the major mutation being G to T transversion [21,22]. TLS over dG-C8-ABP in two different sequences (CCG*GAGGC and CCGGAG*GCC, G* = dG-C8-ABP), which represent codon 248 and 249 sequences of the human p53 tumor suppressor gene, respectively, has confirmed that codon 248 is a hot spot for adduct formation and G to T mutation. These results suggest that the efficiency of TLS over dG-C8-ABP is affected by the surrounding DNA sequences of the ABP lesion, consequently the B/S conformational heterogeneity as described here. Elucidation of conformation-specific bypass, mutational and repair processes over the ABP adducts in the cell should clarify the molecular mechanisms underlying ABP-induced mutagenesis and carcinogenesis.

In the present paper, we demonstrated the combination of SPR binding and HPLC steady-state kinetics as a power tool in investigating FABP-induced conformational heterogeneity in TLS. This approach can be applied to studying other bulky DNA adducts. 

## Figures and Tables

**Table 1:** Steady-state kinetic parameters for insertion of dCTP opposite unmodified FABP−dG adduct with Kf-exo^−.^

Sequence	K_m_ (µM)	k_cat_ (min^−1^)	k_cat_/K_m_ (µM^−1^·min^−1^)	* f_ins_
FABP-G_1_	23.4 ± 0.01	29.7 ± 0.63	1.27	0.33
Control-G_1_	7.4 ± 0.10	28.8 ± 0.96	3.87	1.00
FABP-G_2_	5.7 ± 0.01	5.5 ± 0.11	0.97	0.24
Control-G_2_	5.8 ± 0.11	23.2 ± 0.70	4.00	1.00

* *f*_ins_= (*k*_cat_/*K*_m_)_modified_/(*k*_cat_/*K*_m_)_unmodified control_.

**Table 2:** SPR binding affinities, *K*_D_ (nM) of unmodified (TG_1_G_2_T) and FABP−dG adducts (TG_1_[FABP]G_2_T and TG_1_G_2_[FABP]T) with Kf-exo^−^ (steady-state affinity analysis) in the binary and ternary systems. Association and dissociation rate constants, *k*_a_ and *k*_d_, in binary system are listed.

Sequence	Binary			*K*_D_ of Ternary (nM)			
	*k*_a_ (1/Ms) × 10^7^	*k*_d_ (1/s)	*K*_D_ (nM)	dCTP	dATP	dGTP	dTTP
85-mer TG_1_*G_2_T	15.90 (0.17) ^#^	0.170 (0.002)	1.050 (0.050)	0.200 (0.060)	1.17 (0.04)	1.08 (0.07)	1.08 (0.08)
85-mer control	9.59 (0.05)	0.840 (0.004)	8.740 (0.030)	0.022 (0.001)	14.90 (5.00)	11.40 (4.20)	5.14 (0.74)
84-mer TG_1_G_2_*T	11.10 (0.07)	0.009 (0.000)	0.086 (0.001)	0.045 (0.006)	0.09 (0.00)	0.11 (0.00)	0.09 (0.01)
84-mer control	6.33 (0.04)	0.030 (0.002)	0.480 (0.030)	0.004 (0.000)	1.39 (0.02)	1.25 (0.01)	0.39 (0.04)

^#^ standard deviation.

## Supplementary Materials

The following are available online. Figure S1: HPLC profile of FABP-modified bio-31mer TGGT template and MALDI-TOF characterization of peak 1 and peak 2; Figure S2: Denaturing gel (15%) profiles of 84-mer and 85-mer ligated oligonucleotides, 31-mer, 53-mer and 54-mer non-ligated oligonucleotides; Figure S3: dATP insertion efficiency results of (a) 8-mer to 9-mer and (b) 11-mer to 12-mer in control, TG_1_*G_2_T-FABP and TG_1_G_2_*T-FABP, respectively, at 10 min; Table S1: Binding net stabilization energy of unmodified and FABP-adducts with Kf-exo^−^ in binary system (1:1 binding).
