# Supplementary material for: Probing the Effect of Bulky Lesion-Induced Replication Fork Conformational Heterogeneity Using 4-Aminobiphenyl-Modified DNA
Source: Molecules. 2019 Apr 20;24(8):1566. doi: 10.3390/molecules24081566 (PMC6514942; doi:10.3390/molecules24081566)
Supplement: Supplementary file 1 [file molecules-24-01566-s001.pdf]

Supplemental Information

Probing the Effect of Bulky Lesion-Induced Replication Fork Conformational Heterogeneity Using 4-Aminobiphenyl-Modified DNA

Ang Cai, Ke Bian, Fangyi Chen, Qi Tang, Rachel Carley, Deyu Li,* and Bongsup Cho*

A.C. and K.B. contributed equally to this work.

*Co-correspondence: bcho@uri.edu, Tel: +1 401-874-5024; deyuli@uri.edu, Tel: +1 401-874-9361

Table of Contents

Figure S1. HPLC profile of FABP-modified bio-31mer TGGT template and MALDI-TOF characterization of peak 1 and peak 2. .... S2

Figure S2. Denaturing gel (15%) profiles of 84-mer and 85-mer ligated oligonucleotides, 31-mer, 53-mer and 54-mer non-ligated oligonucleotides. S3

Figure S3. dATP insertion efficiency results of (a) 8-mer to 9-mer and (b) 11-mer to 12-mer in control, $TG_1^*G_2T$-FABP and $TG_1G_2^*T$-FABP, respectively, at 10 min. S3

Table S1. Binding net stabilization energy of unmodified and FABP-adducts with Kf-exo⁻ in binary system (1:1 binding). S4

(a)

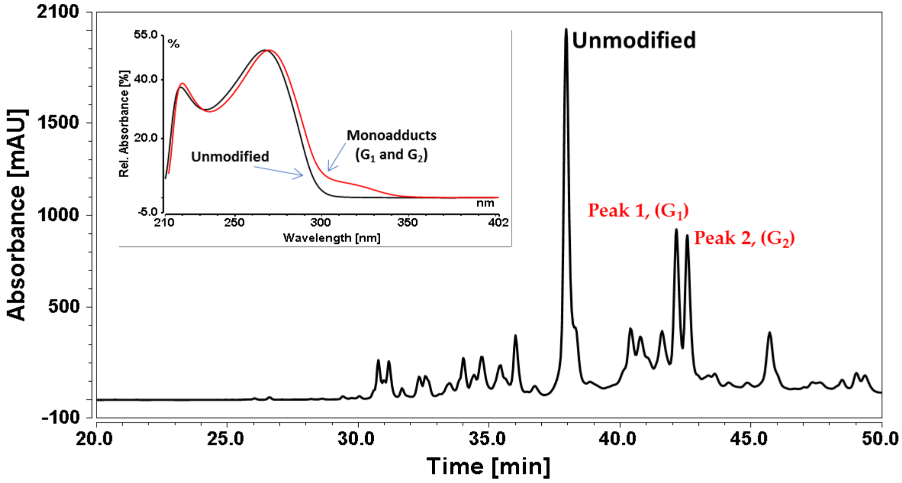

(b)

mass	sequence
9803.1	Bio-CCTCTTCCCTCACCTCTTCTG$_1$G$_2$TCCTCATTC (31mer)
9513.9	Bio-CCTCTTCCCTCACCTCTTCTG$_1$G$_2$TCCTCATT
9209.7	Bio-CCTCTTCCCTCACCTCTTCTG$_1$G$_2$TCCTCAT
8905.5	Bio-CCTCTTCCCTCACCTCTTCTG$_1$G$_2$TCCTCA
8592.3	Bio-CCTCTTCCCTCACCTCTTCTG$_1$G$_2$TCCTC
8303.1	Bio-CCTCTTCCCTCACCTCTTCTG$_1$G$_2$TCCT
7998.9	Bio-CCTCTTCCCTCACCTCTTCTG$_1$G$_2$TCC
7709.7	Bio-CCTCTTCCCTCACCTCTTCTG$_1$G$_2$TC
7420.6	Bio-CCTCTTCCCTCACCTCTTCTG$_1$G$_2$T
7116.4	Bio-CCTCTTCCCTCACCTCTTCTG$_1$G$_2$
6787.3	Bio-CCTCTTCCCTCACCTCTTCTG$_1$ (21mer)
6271.1	Bio-CCTCTTCCCTCACCTCTTCT

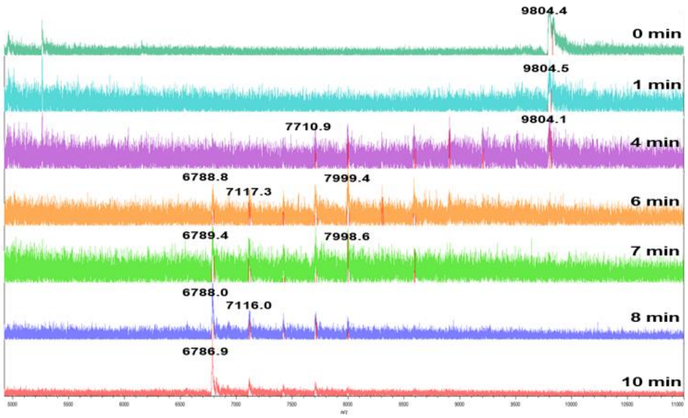

(c)

mass	sequence
9803.1	Bio-CCTCTTCCCTCACCTCTTCTG$_1$G$_2$TCCTCATTC (31mer)
9513.9	Bio-CCTCTTCCCTCACCTCTTCTG$_1$G$_2$TCCTCATT
9209.7	Bio-CCTCTTCCCTCACCTCTTCTG$_1$G$_2$TCCTCAT
8905.5	Bio-CCTCTTCCCTCACCTCTTCTG$_1$G$_2$TCCTCA
8592.3	Bio-CCTCTTCCCTCACCTCTTCTG$_1$G$_2$TCCTC
8303.1	Bio-CCTCTTCCCTCACCTCTTCTG$_1$G$_2$TCCT
7998.9	Bio-CCTCTTCCCTCACCTCTTCTG$_1$G$_2$TCC
7709.7	Bio-CCTCTTCCCTCACCTCTTCTG$_1$G$_2$TC
7420.6	Bio-CCTCTTCCCTCACCTCTTCTG$_1$G$_2$T
7116.4	Bio-CCTCTTCCCTCACCTCTTCTG$_1$G$_2$ (22mer)
6600.1	Bio-CCTCTTCCCTCACCTCTTCTG$_1$
6271.1	Bio-CCTCTTCCCTCACCTCTTCT

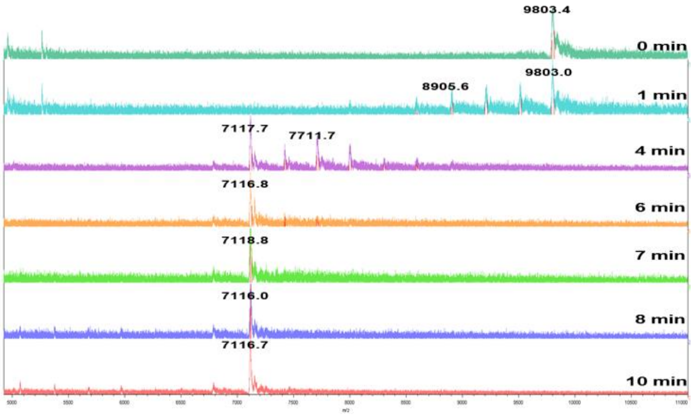

Figure S1. HPLC profile of FABP-modified bio-31mer TGGT template and MALDI-TOF characterization of peak 1 and peak 2. (a) HPLC chromatogram of a reaction mixture of biotinylated-31-mer sequence (5'-Bio-CCTCTTCCCTCACCTCTTCTG$_1$G$_2$TCCTCATTC-3') with an activated **FABP** (*N*-Acetoxy-*N*-(trifluoroacetyl)-4′-fluoro-4-aminobiphenyl) and photodiode array UV spectra of unmodified and mono-adducts. (b) and (c): MALDI-TOF mass spectra of **FABP** modified biotinylated-31mer-TGGT. (b) 3′-Exonuclease digestions of **peak 1** at 0, 1, 4, 6, 7, 8 and 10min. (c) 3′-Exonuclease digestions of **peak 2** at 0, 1, 4, 6, 7, 8 and 10min. Insets show the theoretical MW of the corresponding fragments that should form after 3′-exonuclease digestion.

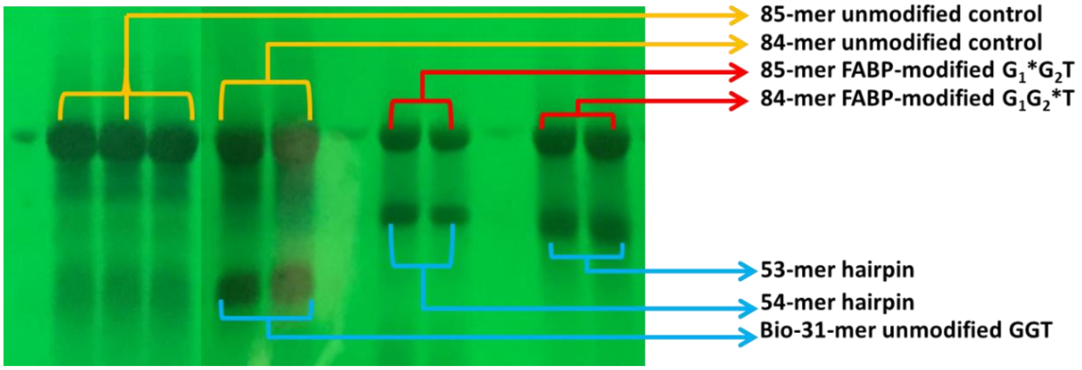

Figure S2. Denaturing gel (15%) profiles of 84-mer and 85-mer ligated oligonucleotides, 31-mer, 53-mer and 54-mer non-ligated oligonucleotides.

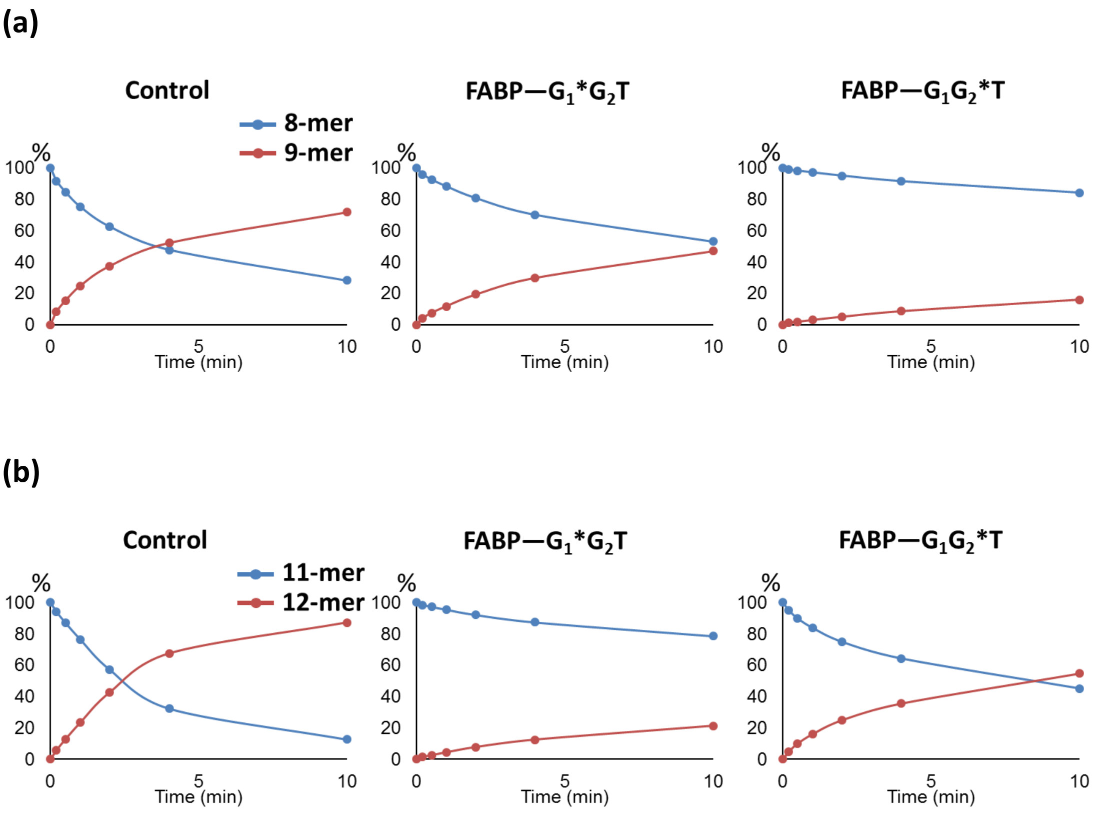

Figure S3. dATP Insertion efficiency of (a) 8-mer to 9-mer and (b) 11-mer to 12-mer in control, TG$_1$*G$_2$T-FABP and TG$_1$G$_2$*T-FABP, respectively, at 10 min (see Figure 3 for sequences).

Table S1. Binding net stabilization energy of unmodified and FABP-adducts with Kf-exo⁻ in binary system (1:1 binding).

Sequence	k_d (1/s)	*Net stabilization Energy (*k*cal/mol)
85-mer control	0.84 (0.004)	0.00
85-mer TG_1[FABP]G_2T	0.17 (0.002)	0.95
84-mer control	0.03 (0.002)	0.00
84-mer TG_1G_2[FABP]T	0.009 (0.0001)	0.71

*Net stabilization energy (*k*cal/mol)= $-RTln(k_d)_{modified} - [-RTln(k_d)_{unmodified}]$
